# Preterm Birth, Age at School Entry and Long Term Educational Achievement

**DOI:** 10.1371/journal.pone.0155157

**Published:** 2016-05-17

**Authors:** David Odd, David Evans, Alan Emond

**Affiliations:** 1 Neonatal Unit, North Bristol NHS Trust, Bristol, United Kingdom; 2 Centre for Child and Adolescent Health, University of Bristol, Bristol, United Kingdom; Hunter College, UNITED STATES

## Abstract

**Objective:**

To investigate if the detrimental impact of year of entering education in preterm infants persists into adolescence.

**Background:**

Preterm infants are often enrolled in school a year earlier than would be expected if this decision is based on their actual date of birth rather than their due date. Initially these infants appear to do disproportionately worse than those who do not ‘skip’ a year. However, it is unclear if this effect remains as the infants grow, to have an important effect on long term achievements in education.

**Design:**

A cohort study, drawn from the Avon Longitudinal Study of Parents and Children (ALSPAC). The exposure measurement was gestational age (defined as preterm (<37 weeks gestation) or term (37–42 weeks)). The primary outcome was a low score at the Key Stage 4 (KS4) educational assessment or receiving special educational needs support (both at age 16). We derived conditional regression models matching preterm to term infants on their date of birth (DOB), their expected date of delivery (EDD), or their expected date of delivery and year of school entry.

**Results:**

After matching for DOB, preterm infants had an increased odds of SEN (OR 1.57 (1.33–1.86)) and the association remained after adjusting for potential confounders (OR 1.39 (1.14–1.68)). The association remained in the analysis matching for EDD (fully adjusted OR 1.43 (1.17–1.74)) but attenuated after restricting to those infants who were enrolled in school in the same year as the control infants (fully adjusted OR 1.21 (0.97–1.52)). There was less evidence for an impact of prematurity on the KS4 score (Matched for DOB; OR 1.10 (0.91 to 1.34), matched for EDD OR 1.17 (0.96 to 1.42) and EDD and same year of schooling, OR 1.00 (0.80 to 1.26)).

**Conclusions:**

This modifiable effect of going to school a year earlier than predicted by their due date appears to have measurable consequences for ex-preterm infants in adolescence and is likely to limit adulthood opportunities.

## Introduction

It is clear that infants born preterm have worse outcomes at school age, including cognitive ability and educational performance[1,2]. There is increasing evidence that the impact is proportionate to the degree of prematurity[3,4], but while neurological injury is commonly seen in extremely preterm infants it is more difficult to identify in those infants born only a few weeks early. Indeed there is evidence that other social factors may exacerbate the impact of prematurity on these infants ability to thrive, in part by a lack of recognising their premature birth[5,6].

In the UK, children are allocated a place at school based on their date of birth and consequently many preterm infants attend school a year earlier than if they were enrolled based on their expected date of birth. All infants who are 4 years old on the 1^st^ of September are allocated a place in reception class at school, and so the age range of the intake ranges from 4 years 0 months to 4 years 11months. Our previous work has suggested that infants placed in a school year prior to the expected one because of their prematurity appear to do disproportionately worse than those who do not ‘skip’ a year[6]. While preterm infants remain at high risk of school failure[2], delaying school entry may be a simple process to improve educational outcomes in this high risk group. Increased flexibility in the system is due to be implemented in some regions soon and consequently some parents of preterm infants will have an opportunity to decide if their child should be enrolled in the school year of their expected date of birth or their actual birth date. However delaying school entry has other important impacts on families and infants and if the early school entry has an important impact on final educational achievement, and hence adulthood opportunities is unknown. The aim of this work is to investigate if the detrimental impact of year of education persists as the child grows into adolescence.

## Methods

The cohort was derived from the Avon Longitudinal Study of Parents and Children (ALSPAC), a longitudinal study based in Bristol, England from April 1991 to December 1992[7] and includes data on over 14,000 infants. Further information about the study can be found on the ALSPAC website: www.alspac.bristol.ac.uk. Methodology was similar to our previous published work[6]. In brief: data on gestational age were derived from the clinical notes and if recorded as less than 37 weeks then was confirmed by reviewing the clinical records. Educational measures were obtained though linkage to the mandatory UK educational assessments, which is split into four stages, with examinations at the end of each stage; Key stage one (KS1) (ages 5–7 years), Key stage two (KS2) (ages 7–11 years), Key stage 3 (KS3) (ages 11–14) and Key stage 4 (KS4) (ages 14–16 years). Tests are applied to all children at the end of these periods.

Governmental standards set the minimum standard expected at each of the first three stages and this was used as the cut-off for a low score. At the end of KS4 children take their school exams, and an *a-priori* cut-off of 5 General Certificates of Secondary Education (GCSE) or equivalent at the A* to C level was used to define a normal score at this age. In addition, children identified as having special educational needs (SEN) in KS4 were identified from the Pupil Level Annual School Census (PLASC). Primary outcomes were therefore: obtaining less than 5 GCSE passes at A*to C level, and being identified as having special educational needs during KS4.

The following perinatal and social factors recorded for the infants were used as confounders of the association between premature birth and the primary outcomes:

- Social factors: Maternal age, socioeconomic group[8] and education and ethnicity.
- Antenatal factors: Gender, parity, weight, length and head circumference at birth.
- Intrapartum factors: Mode of delivery and maternal hypertension.

The dataset contained information on 13,991 infants born alive at between 23 weeks and 42 weeks of gestation. Infants were defined as preterm (less than 37 weeks, n = 898) or term (37–42 weeks, n = 13,093). A total of 1405 infants had none of the outcome measures available, leaving 12,586 infants. As not all infants had all outcome data analyses contained slightly different numbers of children.

Initially we assessed the differences between those infants with outcome data and those without, then the population was split by their gestational age group and their antenatal, social and intrapartum characteristics described.

In the initial analysis, each preterm infant was randomly matched with up to 10 term infants with a date of birth (DOB) within the same calendar month. Any association between gestational age group and school performance was assessed using conditional regression models (with robust standard errors) using outcome and exposure measures as binary variables. Adjustment for possible confounders was performed by adding the potential confounders to the regression models, in the blocks of common variables defined above (e.g. social factors). A multiple imputation data technique (Chained Equations) was used to minimise any potential selection bias in the multivariable models, and allow us to report on the same number of subjects for crude and adjusted analyses[9]. These models were derived using all the variables presented in this paper (including exposure and outcome variables). However each analysis was limited to infants with gestational age and the appropriate outcome measure (i.e. imputed outcome values were not used). Further details of the imputation method are available in S1 Table.

The analysis was then repeated a further two times. In the second analysis infants were matched by their expected date of delivery (EDD) (as opposed to their actual date of birth) and in the third analysis they were matched by their EDD and their year of school attendance (predicted on the child’s date of birth). This last analysis was weighted, to represent the initial cohort using inverse probability weights (rather than bias it to less preterm infants). To assess impact of the school year on educational outcomes, we calculated population attributable risk fractions using the odds ratio from the final adjusted model and the initial population prevalences[10].

Finally, two sensitivity analyses were performed. In one we repeated the conditional regression, but this time splitting exposure into three categories; very preterm (<32 weeks), moderate preterm (32–36 weeks) and term (37–42 weeks). In the second we assessed if the association between year of education and school performance was modified by gender. All analyses were conducted with Stata 10 (Stata Corp, TX, USA). All results are presented as odds ratio (OR) (95% confidence interval (CI)), mean (SD), median (interquartile range (IQR)), or number (percent [%]).

The ALSPAC study was initially given ethical approval from the Local Research Ethics Committees: Bristol and Weston Health Authority (E1808 1989), Southmead Health Authority (49/89 1990) and Frenchay Health Authority (90/8 1990). Prior to enrolment in the ALSPAC study written informed consent was obtained from the mother. For this secondary analysis of data, ethical approval was obtained from the ALSPAC Ethics and Law Committee (ALEC) and approved by the ALSPAC Executive Committee.

## Results

### Sample

The derived cohort is identical the our previous work[6]: with the median gestation in the preterm group was 35 (33–36) weeks, compared to 40 (39–41) weeks in the term group. Infants born preterm had lower birthweights, lengths and head circumferences, were more likely to need resuscitation after birth and had lower Apgar scores (Table 1, all comparisons p<0.001). They were more likely to be born as multiple births and mode of delivery differed from term infants. The mothers of preterm infants also differed from mothers of term infants. In total 1405 infants had missing data on all outcomes, and were not included in any analysis. They were more likely to have older mothers, from higher social economic groups and more educational qualifications. They were also more likely to be male and receive resuscitation at birth, had lower Apgar scores and had lower gestational ages (S2 Table).

**Table 1:** Characteristics of study population.

Measure	Number with data	Preterm (<37 weeks)**(n = 775)**	Term (37–42 weeks) (n = 11811)	P
**Pre-pregnancy factors**				
Maternal age	12,586	27.5 (4.9)	27.9 (5.0)	0.0247
Maternal socioeconomic group	9,052			0.930
I–Professional		22 (4.3%)	460 (5.5%)	
Ii–Managerial		158 (31.0%)	2,610 (31.0%)	
iiiN–Skilled non-manual		41 (8.1%)	685 (8.0%)	
iiiM–Skilled manual		228 (44.8%)	3729 (43.7%)	
iv—Semi-skilled		49 (9.6%)	863 (10.1%)	
v–Unskilled		11 (2.2%)	196 (2.3%)	
Mother’s highest educational qualification*	11,175			0.005
CSE		170 (26.4%)	2,182 (20.7%)	
Vocational		70 (10.9%)	1,079 (10.2%)	
O Level		205 (31.9%)	3730 (35.4%)	
A Level		137 (21.3%)	2,291 (21.8%)	
Degree		61 (9.5%)	1,250 (11.9%)	
Non-white ethnicity		66 (9.3%)	488 (4.5%)	<0.001
**Antenatal and intrapartum factors**				
Primiparous	11,632	348 (48.7%)	4,804 (44.0%)	0.227
Maternal Hypertension	12,585	105 (13.6%)	406 (3.4%)	<0.001
Multiple birth	12,586	149 (19.2%)	186 (1.6%)	<0.001
**Delivery**	11,465			<0.001
Spontaneous cephalic		427 (58.3%)	8,191 (76.3%)	
Emergency caesarean section		166 (22.7%)	624 (5.8%)	
Elective caesarean section		40 (5.5%)	449 (4.2%)	
Instrumental		62 (8.5%)	1323 (12.3%)	
Breech		37 (5.1%)	146 (1.4%)	
**Infant and post-partum factors**				
Male	12,586	443 (57.2%)	6033 (51.1%)	0.001
Birth Weight (g)	12441	2347 (615)	3456 (485)	<0.001
Birth Length (cm)	9518	47.0 (2.6)	50.8 (2.3)	<0.001
Head Circumference (cm)	9664	32.4 (2.1)	34.9 (1.4)	<0.001
Apgar at 1 minute	11,467	9 (7–9)	9 (8–9)	<0.001
Apgar at 5 minute	11,467	9 (9–10)	10 (9–10)	<0.001
Received resuscitation	11,452	182 (24.9%)	838 (7.8%)	<0.001

Standard deviations are given for means of normally distributed continuous variables and percentages for proportions.

* CSE = Certificate in Secondary Education (commonly taken at 16 years of age); Vocational = City & Guilds (intermediate level), technical, shorthand or typing, or other qualification; O level = Ordinary level (commonly taken at 16 years of age); A level = Advanced level (commonly taken at 18 years of age), state enrolled nurse, state registered nurse, City & Guilds (final or full level) or teaching qualification; Degree = University degree

### Outcomes

**Fig 1:**
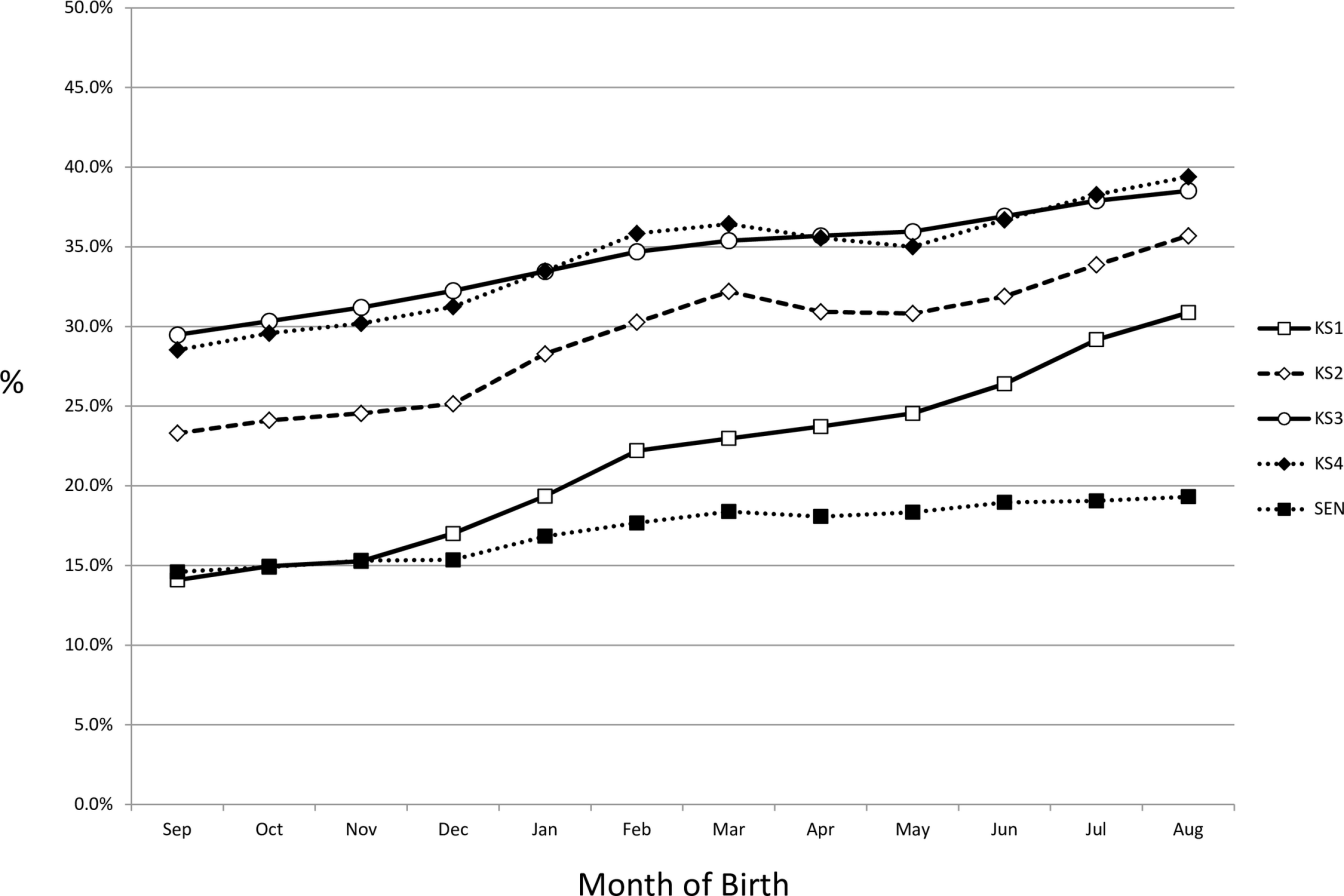
Proportion of children failing Key Stages 1–4 and requiring SEN in KS4, by month of birth. (N.B. School entry based on age on the 1^st^ of September)

Fig 1 shows the risk of a low score at KS1-4 and having SEN, split by gestation group. At all four measurements, infants born later in the school year performed worse, although the effect appeared to attenuate as the children progressed through their education. The increased risks for a poor score, for each month born after September were: KS1 1.6% (1.4%-1.9%), KS2 1.2% (0.9%-1.4%), KS3 0.8% (0.6%-1.1%), KS4 1.0% (0.8%-1.2%), SEN 0.6% (0.4%-0.8%); all p<0.001. Preterm infants had lower KS1-4 scores and higher risk special educational needs during KS4 than term infants (all p<0.01) (Table 2).

**Table 2:** Educational Measures, and Special Educational Needs, split by gestation.

Measure	Number with data	Preterm (<37 weeks)	Term (37–42 weeks)	P
Low KS1 score	10,869	210 (31.7%)	2,171 (21.3%)	<0.001
Low KS2 score	11,499	239 (35.4%)	3,115 (28.8%)	<0.001
Low KS3 score	10,403	251 (39.8%)	3323 (34.0%)	0.003
Low KS4 score	11,405	276 (39.4%)	3610 (33.7%)	0.002
Special educational needs (KS4)	11,100	166 (24.3%)	1737 (16.7%)	<0.001

Measures are mean scores (SD), or number (%) as appropriate

The logistic regression results are shown in Table 3. At KS1 level, after adjusting for potential confounders, preterm infants did worse than their peers when we matched for DOB (OR 1.44 (1.17–1.77)) or EDD (OR 1.53 (1.24–1.88), but not if the analysis was restricted to the same year of schooling (OR 1.26 (0.99–1.60)). At KS2 a similar profile was seen with the measure attenuating between the three models. At KS3 and KS4 there was little evidence for an impact of prematurity in any of the adjusted analyses. However when using SEN as the outcome measure a similar attenuating profile is seen (Matched for DOB; OR 1.39 (1.14–1.68) vs EDD and same year of schooling, OR 1.21 (0.97–1.52)).

**Table 3:** Association between being born preterm (<37 weeks) and school performance.

Measure	Unadjusted	Adjusted for social factors*	Adjusted for social factors and antenatal factors*†	Fully adjusted*†‡
KS1				
Matched for DOB	1.65 (1.38–1.96)	1.57 (1.31–1.88)	1.49 (1.22–1.82)	1.44 (1.17–1.77)
Matched for EDD	1.77 (1.48–2.10)	1.67 (1.40–2.00)	1.59 (1.30–1.95)	1.53 (1.24–1.88)
Matched for EDD and same year of schooling	1.47 (1.19–1.81)	1.38 (1.11–1.71)	1.30 (1.02–1.64)	1.26 (1.00–1.60)
KS2				
Matched for DOB	1.29 (1.09–1.52)	1.22 (1.02–1.46)	1.23 (1.01–1.48)	1.20 (0.99–1.46)
Matched for EDD	1.38 (1.17–1.64)	1.29 (1.08–1.55)	1.27 (1.05–1.54)	1.23 (1.01–1.50)
Matched for EDD and same year of schooling	1.13 (0.93–1.37)	1.05 (0.86–1.29)	1.03 (0.83–1.28)	1.03 (0.82–1.28)
KS3				
Matched for DOB	1.28 (1.08–1.51)	1.19 (1.00–1.43)	1.17 (0.97–1.42)	1.11 (0.91–1.35)
Matched for EDD	1.30 (1.09–1.55)	1.22 (1.02–1.47)	1.20 (0.99–1.46)	1.16 (0.95–1.42)
Matched for EDD and same year of schooling	1.21 (0.99–1.48)	1.10 (0.89–1.36)	1.07 (0.85–1.35)	1.04 (0.82–1.32)
KS4				
Matched for DOB	1.23 (1.05–1.44)	1.15 (0.97–1.37)	1.15 (0.95–1.39)	1.10 (0.91 to 1.34)
Matched for EDD	1.27 (1.08–1.50)	1.16 (0.97–1.39)	1.17 (0.97–1.41)	1.17 (0.96 to 1.42)
Matched for EDD and same year of schooling	1.14 (0.95–1.36)	1.03 (0.84–1.26)	1.01 (0.81–1.25)	1.00 (0.80 to 1.26)
Special educational needs (KS4)				
Matched for DOB	1.57 (1.33–1.86)	1.49 (1.25–1.77)	1.39 (1.15–1.68)	1.39 (1.14–1.68)
Matched for EDD	1.64 (1.39–1.93)	1.54 (1.29–1.83)	1.44 (1.18–1.74)	1.43 (1.17–1.74)
Matched for EDD and same year of schooling	1.40 (1.15–1.70)	1.31 (1.07–1.61)	1.20 (0.97–1.50)	1.21 (0.97–1.52)

* Adjusted for ethnicity, maternal education, socio-economic group and age.

† Further adjusted for gender, parity, weight, length and head circumference at birth.

‡ Further adjusted for mode of delivery and maternal hypertension

Measures are OR (95% CI) for preterm infants vs. term infants

The year of school entry appeared to modify the association between gestational age and the risk of a low KS1 score (p_interaction_ = 0.036), KS2 score (p_interaction_ = 0.002), SEN (p_interaction_ = 0.043), but not KS3 (p_interaction_ = 0.304) or KS4 (p_interaction_ = 0.158).

The population attributable risk fraction for a low KS4 score in the DOB matched analysis was 0.92%, in the EDD matched analysis was 1.47% and in the EDD and school year matched analysis was 0.00%. The population attributable risk fraction for a SEN score in the DOB matched analysis was 3.44%, in the EDD matched analysis was 3.73% and in the EDD and school year matched analysis was 1.94%.

### Sensitivity Analysis

Dividing the preterm group into two sub-groups produced compatible results to the main analysis (Table 4). Very preterm infants had increased risk of a low KS4 score (fully adjusted OR 1.84 (1.20–2.96)) in the initial analysis, which persisted in the EDD matched model but attenuated substantially when restricting to the same year of schooling (fully adjusted OR 1.63 (0.95–2.78)). The association between very preterm infants and special educational needs showed fewer differences across the analyses (fully adjusted results; DOB matched: OR 1.76 (1.06–2.95) vs. EDD and school year: OR 1.78 (1.05–3.02)). Infants born moderately preterm also showed little evidence of increased risk of a low KS4 score (fully adjusted; DOB 1.05 (0.85–1.30) vs. EDD and school year OR 0.93 (0.73–1.19)) although effect on special educational needs attenuated through the 3 analyses (fully adjusted; DOB matched: OR 1.27 (1.03–1.58) vs. EDD and school year: OR 1.15 (0.90–1.46)). While male infants had a higher risk of a low KS4 score (e.g. DOB adjusted analysis: OR 1.83 (1.62–2.06)), there was little evidence that this was differentially worse for boys in the wrong school year (EDD and school year matched: OR 1.82 (1.66–2.00), p_interaction_ = 0.691)

**Table 4:** Association between being born very or moderate preterm and school performance.

Measure	Very preterm(<32 weeks)		Moderate preterm(32–36 weeks)	
	Unadjusted	Fully adjusted*†‡	Unadjusted	Fully adjusted*†‡
Poor outcome at KS1				
Matched for DOB	2.45 (1.67–3.60)	2.26 (1.49–3.42)	1.48 (1.22–1.81)	1.29 (1.02–1.62)
Matched for EDD	2.69 (1.85–3.91)	2.34 (1.55–3.54)	1.58 (1.29–1.93)	1.37 (1.09–1.72)
Matched for EDD and same year of schooling	1.88 (1.10–3.21)	1.59 (0.89–2.84)	1.41 (1.12–1.77)	1.22 (0.94–1.57)
Poor outcome at KS2				
Matched for DOB	1.97 (1.35–2.87)	1.81 (1.19–2.75)	1.17 (0.97–1.41)	1.10 (0.89–1.36)
Matched for EDD	2.20 (1.49–3.26)	1.86 (1.18–2.92)	1.24 (1.03–1.50)	1.12 (0.90–1.39)
Matched for EDD and same year of schooling	1.82 (1.14–2.91)	1.55 (0.90–2.69)	1.06 (0.86–1.30)	0.97 (0.76–1.23)
Poor outcome at KS3				
Matched for DOB	2.11 (1.43–3.13)	1.86 (1.17–2.95)	1.14 (0.94–1.37)	0.99 (0.80–1.22)
Matched for EDD	2.11 (1.41–3.16)	1.91 (1.18–3.07)	1.16 (0.96–1.41)	1.05 (0.84–1.30)
Matched for EDD and same year of schooling	2.23 (1.31–3.79)	2.00 (1.07–3.73)	1.10 (0.89–1.37)	0.95 (0.73–1.23)
Poor outcome at KS4				
Matched for DOB	1.74 (1.22–2.50)	1.84 (1.20–2.83)	1.13 (0.94–1.35)	1.05 (0.85–1.30)
Matched for EDD	1.89 (1.30–2.75)	1.84 (1.20–2.83)	1.16 (0.96–1.39)	1.05 (0.85–1.31)
Matched for EDD and same year of schooling	1.88 (1.18–3.02)	1.63 (0.95–2.78)	1.05 (0.86–1.29)	0.93 (0.73–1.19)
Special educational needs (KS4)				
Matched for DOB	2.09 (1.43–3.06)	1.76 (1.06–2.95)	1.46 (1.21–1.76)	1.27 (1.03–1.58)
Matched for EDD	2.10 (1.48–2.99)	1.90 (1.26–2.87)	1.53 (1.27–1.85)	1.33 (1.06–1.65)
Matched for EDD and same year of schooling	1.84 (1.14–2.98)	1.78 (1.05–3.02)	1.34 (1.09–1.66)	1.15 (0.90–1.46)

* Adjusted for ethnicity, maternal education, socio-economic group and age, gender, parity, weight, length and head circumference at birth, mode of delivery and maternal hypertension

Measures are OR (95% CI) for preterm infants vs. term infants

## Discussion

In this study we have shown that the impact of prematurity identified in our previous work[6] persists into later school performance. In adolescence, ex-preterm infants still have a higher chance of having special educational needs than their term peers, but this association is weakened if the infant is placed in the correct school year. Overall we saw only a weak impact on the overall educational results, but in a sensitivity analysis, infants born below 32 weeks remained at higher risk of a poor school outcome, and the effect was also attenuated by the year of schooling.

The population impact of prematurity showed a similar profile, suggesting that early school attendance of preterm infants accounts for 1 out of every 60 infants who need special educational needs in the later stages of their education.

As before, the strength of this work is that it is based on a population based cohort study with prospectively collected data on many important confounders. Like many studies of its type its main limitation is related to that of missing data. A total of 14% of the eligible cohort had no outcome data and hence were excluded from all analyses. This potential selection bias is a limitation which needs to be considered when interpreting the results presented here. We did however use a multiple imputation technique in both this and our previous work to reduce the impact of missing confounders and maximise the data utility[6]. One further limitation is that we have also assumed that infants entered schooling in the year that they were offered a place. While standard practice, it may be that some parents of preterm infants successfully lobbied for a delayed entry into school and if so this would likely lead us to underestimate the true effect size of being in the ‘wrong’ year group.

It should also be noted that the infants included in this work were born 20 years ago. This does of course allow analysis of their longer term educational outcomes, but any changes in educational and medical care in this time will not be reflected in this work: however, school failure in still a major concern after preterm birth[11,12].

It is important to note while the effect of prematurity did attenuate as the children grew, it was also confounded by factors related to the cause of the prematurity. Indeed, the apparent paradoxical effect of a bigger impact when we matched with EDD vs DOB may be explained due to matching increasing numbers of preterm infants with term peers in the next school year and demonstrates the difficulty in measuring the true impact of prematurity on complex outcomes like education. While this work confirms the increased risk of a poor educational outcome for boys, this effect did not appear to be exacerbated by entering school a year early. The attenuation of the impact on educational outcomes in this older group of children who were born preterm may be due to early recognition and support of additional needs (as evidenced by their increase risk of SEN). However, other effects on self-esteem and social interaction seem likely. Overall, we can conclude that delayed school entry, to their ‘correct’ school year, would benefit this cohort of infants: particularly those born extremely preterm

Recent proposed changes in legislation in the UK may mean that many parents will be offered flexibility over the school start date of summer-born infants. For some preterm infants this will provide the opportunity to start in the school year defined by their expected date of birth and our results suggest that this may well lead to improved late educational outcomes. In addition, if the reduction in special educational needs is replicated, this ‘later’ entry into school is likely to result in significant financial savings for education budgets. However the level of support and nursery provision that will be provided for the extra year if parents choose to delay school entry is currently unclear, and will be vital if this change in education policy is to benefit these ex-preterm children.

## Conclusions

This work shows that despite 10 years of education, and while the impact of prematurity appears to attenuate as children grow, preterm infants remain at higher risk of low GCSE scores and needing special educational support. Importantly the easily modifiable effect of going to school in a year earlier than predicted by their due date appears to still have measurable consequences for ex-preterm infants in adolescence, and consequently is likely to limit adulthood opportunities. This work supports the need for flexibility on the age of admission to school for this group, with potential educational benefits to the infants and financial benefits to the education service.

## Supporting Information

S1 Table Details of Multiple Imputation Methods. All variables presented in the paper (including exposure and outcome variables) were included in the imputation model. Analysis was based on 20 imputed datasets.
(DOCX)

S2 Table Characteristics of infants with missing outcome data. Standard deviations are given for means of normally distributed continuous variables and percentages for proportions. * CSE = Certificate in Secondary Education (commonly taken at 16 years of age); Vocational = City & Guilds (intermediate level), technical, shorthand or typing, or other qualification; O level = Ordinary level (commonly taken at 16 years of age); A level = Advanced level (commonly taken at 18 years of age), state enrolled nurse, state registered nurse, City & Guilds (final or full level) or teaching qualification; Degree = University degree.
(DOCX)
