# Supplementary material for: Preterm Birth, Age at School Entry and Long Term Educational Achievement
Source: PLoS One. 2016 May 17;11(5):e0155157. doi: 10.1371/journal.pone.0155157 (PMC4871348; doi:10.1371/journal.pone.0155157)
Supplement: S1 Table — All variables presented in the paper (including exposure and outcome variables) were included in the imputation model. Analysis was based on 20 imputed datasets. (DOCX) [file pone.0155157.s001.docx]

S1 Table. Details of Multiple Imputation Methods

All variables presented in the paper (including exposure and outcome variables) were included in the imputation model. Analysis was based on 20 imputed datasets.

| **Imputation Variable** | **n** | **%** | **Imputation Command** |
| --- | --- | --- | --- |
| Gender | 0 | 0.0% | - |
| Gestation | 0 | 0.0% | - |
| Multiple Birth | 0 | 0.0% | - |
| Maternal Age | 0 | 0.0% | - |
| Maternal Socioeconomic Status | 3,534 | 28.1% | ordinal |
| Maternal Education | 1,411 | 11.2% | ordinal |
| Parity | 954 | 7.6% | logistic |
| Ethnicity | 998 | 7.9% | logistic |
| Mode of delivery | 1,121 | 8.9% | multinomial |
| Hypertension | 0 | 0.0% | - |
| Neonatal Resuscitation | 1,134 | 9.0% | logistic |
| Birth weight | 145 | 1.2% | linear |
| Birth length | 3,068 | 24.4% | linear |
| Birth head circumference | 2,923 | 23.2% | linear |
| Incorrect schooling year | 0 | 0.0% | - |
| Special Educational Needs | 1,486 | 11.8% | Logistic |
| Key Stage 1 summary score | 1,722 | 13.7% | Linear |
| Low Key Stage 1 score | 1,717 | 13.6% | Logistic |
| Key Stage 2 summary score | 1,103 | 8.8% | Linear |
| Low Key Stage 2 score | 1,087 | 8.6% | Logistic |
| Key Stage 3 summary score | 3,137 | 24.9% | Linear |
| Low Key Stage 3 score | 2,183 | 17.3% | Logistic |
| Key Stage 4 summary score | 1,181 | 9.4% | Linear |
| Low Key Stage 4 score | 1,181 | 9.4% | Logistic |
| Incorrect Year of Schooling*Preterm | 0 | 0.0% | - |
