# Supplementary material for: Preterm Birth, Age at School Entry and Long Term Educational Achievement
Source: PLoS One. 2016 May 17;11(5):e0155157. doi: 10.1371/journal.pone.0155157 (PMC4871348; doi:10.1371/journal.pone.0155157)
Supplement: S2 Table — Standard deviations are given for means of normally distributed continuous variables and percentages for proportions. * CSE = Certificate in Secondary Education (commonly taken at 16 years of age); Vocational = City & Guilds (intermediate level), technical, shorthand or typing, or other qualification; O level = Ordinary level (commonly taken at 16 years of age); A level = Advanced level (commonly taken at 18 years of age), state enrolled nurse, state registered nurse, City & Guilds (final or full level) or teaching qualification; Degree = University degree. (DOCX) [file pone.0155157.s002.docx]

**S2 Table. Characteristics of infants with missing outcome data**

| **Measure** | **Number with data** | **Outcome data available**  **(n=12586)** | **Missing outcome data**  **(n=1405)** | **P** |
| --- | --- | --- | --- | --- |
| **Pre-pregnancy factors** |  |  |  |  |
| Maternal age | 13991 | 27.9 (4.9) | 28.9 (5.0) | <0.001 |
|  |  |  |  |  |
| Maternal socioeconomic group | 10,058 |  |  | <0.001 |
| I – Professional |  | 482 (5.3%) | 109 (10.8%) |  |
| Ii – Managerial |  | 2768 (30.6%) | 393 (39.1%) |  |
| iiiN – Skilled non-manual |  | 726 (8.0%) | 65 (6.5%) |  |
| iiiM – Skilled manual |  | 3957 (43.7%) | 347 (34.5%) |  |
| iv - Semi-skilled |  | 912 (10.1%) | 78 (7.8%) |  |
| v – Unskilled |  | 207 (2.3%) | 14 (1.4%) |  |
|  |  |  |  |  |
| Mother’s highest educational qualification* | 12,402 |  |  | <0.001 |
| CSE |  | 2352 (21.1%) | 149 (12.1%) |  |
| Vocational |  | 1149 (10.2%) | 71 (5.8%) |  |
| O Level |  | 3935 (35.2%) | 367 (29.9%) |  |
| A Level |  | 2428 (21.7%) | 355 (28.9%) |  |
| Degree |  | 1311 (11.7%) | 285 (23.2%) |  |
|  |  |  |  |  |
| Non-white ethnicity | 11,608 | 554 (4.8%) | 2 (10.0%) | 0.275 |
|  |  |  |  |  |
| **Antenatal and intrapartum factors** |  |  |  |  |
| Primiparous | 12,942 | 5152 (44.3%) | 634 (48.4%) | 0.074 |
| Maternal Hypertension | 13,991 | 511 (4.1%) | 56 (4.0%) | 0.893 |
|  |  |  |  |  |
| Multiple birth | 13,991 | 335 (2.7%) | 35 (2.5%) | 0.705 |
|  |  |  |  |  |
| Delivery | 12,711 |  |  | 0.015 |
| Spontaneous cephalic |  | 8618 (75.2%) | 890 (71.4%) |  |
| Emergency caesarean section |  | 790 (6.9%) | 105 (8.4%) |  |
| Elective caesarean section |  | 489 (4.3%) | 52 (4.2%) |  |
| Instrumental |  | 1385 (12.1%) | 169 (13.6%) |  |
| Breech |  | 183 (1.6%) | 30 (2.4%) |  |
|  |  |  |  |  |
| **Infants and post-partum factors** |  |  |  |  |
| Male | 13,991 | 6476 (51.5%) | 766 (54.5%) | 0.029 |
| Birth Weight (g) | 13803 | 3388 (560) | 3325 (690) | <0.001 |
| Birth Length (cm) | 10503 | 50.6 ( 2.5) | 50.5 ( 3.0) | 0.1646 |
| Head Circumference (cm) | 10663 | 34.7 (1.57) | 34.7 (1.9) | 0.2354 |
| Apgar at 1 minute | 11467 | 9 (8-9) | 9 (8-9) | <0.001 |
| Apgar at 5 minute | 11467 | 10 (9-10) | 10 (9-10) | <0.001 |
| Gestation | 12586 | 40 (39-41) | 40 (38-40) | 0.0008 |
| Received resuscitation | 12,666 | 1020 (8.9%) | 147 (12.1%) | <0.001 |
|  |  |  |  |  |

Standard deviations are given for means of normally distributed continuous variables and percentages for proportions.

* CSE=Certificate in Secondary Education (commonly taken at 16 years of age); Vocational=City & Guilds (intermediate level), technical, shorthand or typing, or other qualification; O level=Ordinary level (commonly taken at 16 years of age); A level=Advanced level (commonly taken at 18 years of age), state enrolled nurse, state registered nurse, City & Guilds (final or full level) or teaching qualification; Degree=University degree
